# COVID-19 and the Nigerian child: the time to act is now

**DOI:** 10.11604/pamj.supp.2020.35.23286

**Published:** 2020-06-18

**Authors:** Datonye Christopher Briggs, Tamuno-Wari Numbere

**Affiliations:** 1 Department of Paediatrics, Rivers State University Teaching Hospital, Rivers State, Nigeria; 2 Department of Public Health, Rivers State Ministry of Health, Rivers State, Nigeria

**Keywords:** Nigeria, child, COVID-19, pandemic, SARS-Cov-2, virus, impact

## Abstract

COVID-19 has overwhelmed virtually every sector in resource-rich countries of the world and has gradually but steadily enveloped almost all of Africa. While her leaders grapple with the vivid realization of the myriad effects of the virus, the African children should not be the ‘hidden victims’ of the COVID-19 pandemic because they are among the most vulnerable. This narrative highlights the effects of the pandemic on the economic, education, health, mental and socio-cultural well-being of the Nigerian child and suggests ways to mitigate it. The impact of COVID-19 pandemic on the Nigerian child are numerous. Policies should be set up urgently and interventions sourced to ameliorate the effect of the virus on the most vulnerable group in Nigeria.

## Essay

The world as it is today is being overwhelmed by the outbreak of the novel severe acute respiratory syndrome coronavirus 2 (SARS-Cov-2) virus that is responsible for the COVID-19 disease [1]. The World Health Organization (WHO) declared the new wave of infection with predominantly respiratory system symptoms a pandemic on March 11th, 2020 with most countries reporting increasing numbers of morbidity and mortality rates [2]. The major drivers of the outbreak appear to be both symptomatic and asymptomatic persons infected with SARS-COV-2 from whom the virus can spread via droplets or direct contact with contaminated surfaces [3]. This forcefully and rapidly led to a systematic lockdown of most countries of the world [4]. Since the WHO had recommended a multi-prong preventive approach that includes physical distancing, hand washing with soap and water for at least 20 seconds and respiratory etiquette as mitigating measures, the lockdown eventually became a key method of restricting on-going community spread of the virus [1]. The resultant effects of these stringent public health actions have led to enormous economic losses, disruption of the ‘usual’ physical and social contacts, massive loss of jobs and means of livelihood and increase in mental health issues [5,6]. However, the effects of COVID-19 on the wellbeing of children across Africa is of concern and seems to lag.

Africa in the trend of events appeared to be the last continent hit by the pandemic. While the reports of new cases and death toll (epidemic curve) appears to be flattening and decreasing in other countries of the world [7], this cannot be said among the countries in Africa especially sub-Saharan Africa, where testing for COVID-19 is still a major challenge and healthcare facilities are in deplorable states [8]. Reports from China and other western countries demonstrate that children are largely unaffected and even for those diagnosed COVID-19 positive, severe disease and death is very unusual [9]. However, children are undeniably caught in the predicament COVID-19 is causing and their overall wellbeing risks being overturned due to the impact already ensuing in their families and immediate surroundings. Africa is less likely to consider the impact of COVID-19 on children early in the course of the response, as has been noted in earlier outbreaks [10]. Presently, with the rising incidence of newly diagnosed cases in Africa and more so in Nigeria, the well-being of children could pose a challenge as its leaders grapple with the most pressing needs which include food insecurity and economic burden of the pandemic [11]. The lockdown, therefore, makes children in these resource-limited settings and their families alike the hardest hit by both the virus and the ongoing response [12]. This narrative review highlights the likely impact of COVID-19 on the Nigerian Child under six sub-headings.

**Impact on the economy:** the slowing down in the global economy and lockdown of countries around the world as a result of COVID-19 has taken its toll on the global demand for oil. This drastic decline particularly affects a country like Nigeria whose sole Gross Domestic Product (GDP) and foreign reserves are dependent on foreign trade in oil. The odds, however, are not in the favour of Nigeria as the pandemic exerts pressure on the economy which was already contending with an earlier recession in 2016 [13]. Unfortunately despite an initial projected budget revenue allocation of 8.24 trillion naira, COVID-19 outbreak has had a negative toll on the oil price benchmark and also oil production respectively [14]. At the time of this review, it is about the second month after the first case of COVID-19 was reported in Nigeria and some state governors have already declared their inability to sustain payment of salaries to workers in the public sector [15]. With a high unemployment rate of 23.1%, almost half of the population are living below the poverty line ($2 a day) [16]. Children are thus affected indirectly because with the restrictions on international trade, closure of markets, delay and or non-payment of the non-essential workforce, the pandemic has contributed in no small measure to food scarcity and price inflation in the cost of staple food items in the country hence the level of hunger and poverty are on the increase [11]. The impact is greatest especially among children whose parents or guardians depend on daily income for survival which has been abruptly halted due to the on-going lock down parts in the country. With the already existing alarming rates of child-poverty in Nigeria [17], it is anticipatable that the impoverished would rather prefer the COVID-19 disease than to be hunger-stricken [18]. COVID-19, therefore, has already spun into action a vicious cycle of poverty, hunger and malnutrition in these already vulnerable or at-risk children. Without any urgent interventions, the situation could worsen because the pandemic risks undermining all the earlier efforts to reverse the trend of rising hunger in the country.

**Impact on education:** according to UNICEF about 1.6 billion children and young people are unable to be physically present at school due to the temporary closure of schools that have impacted over 91 per cent of students globally [19]. Although some schools can endeavour to provide online classes, this is unavailable to the majority of children and young people in Africa [20]. Since school closures, many families in Nigeria have found themselves unable to help their wards keep track with their education. Although learning platforms have been launched by UNICEF and Microsoft to aide affected children and young people continue their education at home in other parts of the world [19], this is largely unavailable in resource-limited settings like Nigeria where many students lack computers or high-speed internet services, making a considerable number of families unable to afford or sustain its use as a means for educating their wards [20]. The government in Nigeria, fully aware of these challenges have opted to have daily live teaching sessions on radio and television at scheduled time intervals in the entire country, albeit, there are many more children in semi-urban and rural areas without access to internet services and very limited electric power supply. These children are more disadvantaged and hence underserved because they have no access to formal education in this period. The impact of homeschooling on education, especially in families in Nigeria that lack an organizational structure remains to be seen.

**Impact on health:** the health hazards and human consequences of the COVID-19 is already known and the fact that it has nearly crippled the health workforce and outstretched the health care systems in resource-rich countries of the world is undeniable [21]. In most countries in Sub Saharan Africa including Nigeria, there was already an existent endemic ‘weak healthcare system’ with substandard personnel, infrastructure and funding in the pre-COVID-19 era [22]. Presently, due to the restrictions in movement and lockdown, commuting to the healthcare facilities when children are sick might be delayed. Also, the anxiety associated with exposing a child (and parent) to a potential site that brings them in close contact with the contagion during hospital visits may make parents less likely to present with their sick child early. Other possible challenges include the delay in receiving the urgent care needed if a child with asthma presents to healthcare centres as there is a substantial overlap between the clinical presentation of worsening asthma and COVID-19, and an increasing community spread also lessens the likelihood of obtaining a history of known contact with a confirmed case [23]. It has been reported that screening for COVID-19 is required if available in any asthmatic child who comes to medical attention with worsening cough or shortness of breath [23]. However, in settings where screening tests are not readily available, as is the case in most Nigerian healthcare facilities, undue delays could result in severe or life-threatening asthmatic episodes being misdiagnosed, mismanaged, abandoned or referred because of the fear of contracting the dreaded virus by healthcare workers who are inadequately protected. Similar instances could arise when children present with other viral or atypical types of pneumonia. Furthermore, there is the likelihood of widening the gap already covered by vaccination efforts and making more vulnerable children at risk of dying from vaccine-preventable diseases due to a disruption in immunization scheduled programmes in Nigeria. With an already low vaccine coverage rate of 32% in 2018, even brief interruptions of vaccination activities would only make outbreaks more likely to occur [24]. Presently Nigeria along with other sub-Saharan countries like Chad, Ethiopia and South Sudan are beginning to experience a resurgence of measles because the mass vaccination campaigns have been halted due to the COVID-19 pandemic [25]. Yet another impact will be the possible shortage of drugs/ supplies and challenges with diagnosing high risk/immunosuppressed children for HIV and tuberculosis. With the on-going restriction of movements, children on antiretroviral and anti-tuberculosis medications may face the challenges of stock outs and adherence issues due to the inability of the healthcare facilitates to prescribe medications for longer periods to cover the likely duration of the pandemic to avoid the risk of exposure to the virus [26].

**Impact on mental health:** it is plausible that prolonged physical and social distancing, lockdowns and closure of schools can harm the psychosocial wellbeing of children and young adults [27]. Mental problems ranging from stress to anxiety, depression and sleep disorders have been reported in children in epidemic settings [27]. Children also suffer from the effects of an abusive parent or guardian regularly because there is nowhere to seek help and avenues to let out their frustrations are very limited due to the closure of schools [28]. What happens to children who eventually become COVID-19 positive, get treated, recover and are reintegrated back into their schools and the society remains to be seen.

**Sociocultural impact:** there is also a likely increase in physical and sexual abuse, with children being at increased risk of indecent exposure to pornography, increase in teenage pregnancies, increased occurrences of armed robbery, bullying and outright hooliganism stemming from the prolonged lockdowns, worsening lack of food, inflation in prices of staple foods and poverty [29,30].

**Impact on orphaned and vulnerable children:** these include the street children, almajiris´, child refugees, children in conflict-torn regions, emancipated minors, those with disabilities, in orphanages and correction facilities. These particularly marginalized groups are in precarious situations because of the high rates of poverty, malnutrition, hunger and abuse [29]. Less attention may be given to these group of Nigerian children and they having to cope with COVID-19 pandemic, unaided, is very worrisome.

**Suggested approaches to mitigate the effects of COVID-19 on the Nigerian child:** the possible solutions would ultimately depend on the unique epidemiological context, political will, health care financing and the variations in health service provision patterns in Nigeria. While the government of Nigeria and stakeholders presently grapple with the COVID-19 pandemic as it unravels in varying dimensions daily, they should endeavour to act on each threat and have dynamic and creative strategic approaches to reduce the impact on the children in Nigeria. Children-specific approaches to mitigate the effects of COVID-19 would include: Implementing a coordinated response whereby paediatricians and other allied healthcare workers are set up to be ‘think tanks’ to formulate urgently evidence-based recommendations to: 1) Prioritize provision of meals to support the most vulnerable school-aged children across Nigeria in collaboration with the already existing school health programme schemes in the various states; 2) Prioritize education for every child in Nigeria and seek ways to reach the rural and semi-urban areas without access to television, radio or the internet; 3) Make guidelines on the management of paediatric patients during COVID-19 pandemic which should specifically address isolation facilities for children in healthcare institutions, triaging of children with signs of respiratory tract infections in out-patient-clinics, needed specifications and adjustments for attending deliveries of COVID-19 positive pregnant mothers, special care baby units and the breastfeeding and kangaroo mother care practices, routine immunization services and paediatric out-patient clinics. 4) Make guidelines on infection control specific to the paediatric population. 5) Make guidelines on support needs for the vulnerable and orphaned children including modalities to reach the malnourished and impoverished. 6) Prioritize providing ethical and psychosocial support services for children and families during and after the COVID-19 pandemic. 7) Advocacy with government and inter-sectoral collaboration to formulate appropriate child protection-focused programmes. 8) Provide a paediatric pandemic plan that will inform policymakers through promoting the understanding of COVID-19 and how it affects children: how next to be prepared in the future. 9) Other indirect ways to mitigate the impact in children seeking healthcare will include a surge capacity planning - the ability to scale up delivery of health interventions proportionately for the severity of the disease and population at risk: To urgently escalate COVID-19 testing which should include testing of children across all states. Reactivate her two-way referral system to ensure the more severe cases get tertiary care and probable cases are immediately isolated before the Nigerian Centre for Disease Control is notified and or referral to tertiary or known isolation centres. Massive training of healthcare providers on IPC protocols and ensure basic personal protective equipment are made available to the health centres. Engage the private and or general government hospitals by empowering them to review and manage regular medical and low-risk surgical cases to enable decongesting of the overburdened tertiary centres.

## Competing interests

The authors declare no competing interests.
